# Without Systems and Complexity Thinking There Is no Progress - or Why Bureaucracy Needs to Become Curious Comment on "What Can Policy-Makers Get out of Systems Thinking? Policy Partners’ Experiences of a Systems-Focused Research Collaboration in Preventive Health"

**DOI:** 10.34172/ijhpm.2020.45

**Published:** 2020-04-04

**Authors:** Joachim P. Sturmberg

**Affiliations:** ^1^School of Medicine and Public Health, University of Newcastle, Callaghan, NSW, Australia.; ^2^International Society for Systems and Complexity Sciences for Health, Waitsfield, VT, USA.

**Keywords:** Transformational Leadership, Systems and Complexity Thinking, Design Thinking, ProblemSolving, Health System Redesign

## Abstract

The bureaucracy’s goal is to maintain uniformity and control within discrete areas of activity and relies on hierarchical processes and procedural correctness as means to suppress autonomous decision making. That worldview, however, is unsuited for problem solving of real world VUCA (Volatility, uncertainty, complexity and ambiguity) problems. Solving *wicked* problems in the VUCA world requires curiosity, creativity and collaboration, and a willingness to deeply engage and an ability to painstakingly work through their seemingly contradictory and chaotic pathways. In addition, it necessitates leadership. Leaders require a deep – indeed academic – understanding of the nature of the problems and the veracity of various problem-solving approaches. Leadership after all means "[facilitating] *the necessary adaptive work that needs to be done by the people connected to the problem."* That are the people at the coalface who understand and have to manage the complexities relating to problems unique to their local environment for which *of the shelf solutions* never work. *Systems and complexity thinking* is more than a tool, it is – in a sense – a way of being, namely deeply interested in understanding the highly interconnected and interdependent nature of the issues affecting our life and work. Hence, *system and complexity thinking* is, contrary to what Haynes and colleagues state in their "summation for the public reader," neither* "overwhelming and hard* [nor difficult] *to use practically* ." Such a view is as much misleading as selfdefeating.

*People often say that I’m curious about too many things at once... But can you really forbid a man from harbouring a desire to know and embrace everything that surrounds him*?

Alexander von Humboldt

Was Humboldt defending himself against a bureaucrat when he made this statement?^1^ We will never know; however, it is long clear that bureaucracy has never been known to be particularly curios or enterprising. Bureaucracy is a *closed system* designed to maintain uniformity and control within discrete areas of activity; hierarchical processes and procedural correctness are paramount to suppress autonomous decision-making. Haynes et al^2^ provide a fascinating insight into the worldview of the health bureaucracy and its consequences on achieving urgent change. Their work is particularly helpful as it provides a deeper understanding of the inner workings of health ministries and departments (and most like all other related institutions). Resistance to change is a *build-in systemic feature* of these institutions and involves as much structural as intellectual dimensions.

However, Haynes and colleagues’^2^ instrumental framing – *what can*[one] *get out of systems thinking* – misses a fundamental point, namely that systems and complexity thinking in the first instance are *mental approaches*. They reflect an appreciation that problems are *interconnected* and *interdependent wholes* with *nonlinear dynamics*, hence there cannot be predictably solutions. Indeed, best-possible solutions to such problems can only arise from the continual engagement and adaptation of its stakeholders. This commentary firstly provides a brief background to systems and complexity thinking and its application to problem-solving in an uncertain environment. It then describes the nature of adaptive leadership and highlights that *true* leaders engage in the problem-solving process (rather than simply prescribe *their* solutions). It concludes with a plea – let’s all promote systems and complexity thinking as *a natural and intuitive way* to approach the problems in our constantly changing world.

## Systems and Complexity Thinking – A Different Way of Seeing the World

Max Planck famously said: “*When you change the way you look at things, the things you look at change.*”

Seeing things as *interconnected wholes* results in a different appreciation than seeing *the whole as a sum of its parts*.^3^ Equally, *systems and complexity thinking* fosters a view to understand the structure and function of things based on the interconnection and interactions amongst its building blocks (technically speaking, its agents) whereas reductionist thinking forces a view to understand the structure and function of a thing based on the study of its constituent parts.^4^

Systems – regardless of being mechanical or living – are “*whole*[s]* consisting of two or more parts (1) each of which can affect the performance or properties of the whole, (2) none of which can have an independent effect on the whole, and (3) no subgroup of which can have an independent effect on the whole.*”^5^ In addition organisational systems require a *focus* to orientate themselves and to *stay on track* in a constantly changing environment if they truly want to achieve their *purpose and goals*.^6^

## Changing the Parts Is not Going to Improve the System-as-a-Whole

The reductionist focus on the parts is invariably counterproductive, as simply improving a part of a system is not improving the *system-as-a-whole,* unless the improvement of a part also achieves an improvement of the *system-as-a-whole*. Improvements of parts that do not improve the *system-as-whole* are not worth the effort.^5^ These insights encapsulate the meaning of *systems and complexity thinking* – it is a way of thinking about the particulars in their distinct context and their consequences in time.

This unambiguously means that there are no *of the shelf* solutions – *seemingly the same problem* will have significant different characteristics and dynamics in another context. Every problem is unique, every problem needs a fresh mental approach, and every problem has its own unique solution.

*Designing* ^7^ and *dynamic simulation modelling*^8^ are two common approaches in the nonlinear toolbox frequently used to tackle issues requiring conceptual or policy answers.

The strength of *designing*and *dynamic simulation modelling*lies in their collaborative approach – all associated with the problem are involved, providing reflections and formulating potential solutions. Key is the common ground rule – no contribution is clever or silly and no proposition is right or wrong. These approaches work on the basis of trust, namely that all contributions provide valuable perspectives that help all to learn and further their trust in their ability to collectively find the most adapted solution through effective collaboration.

While these processes may take a little more time, they will invariably lead to better outcomes when confronted with *wicked problems*, ie, issues that are not *completely definable*, have *no definable end*, and have *no one correct solution*. In particular, every solution itself will result in a *new wicked problem*.^9^ That is the VUCA world we live in – we are constantly dealing with volatility, uncertainty, complexity and ambiguity, the only way forward is to respond with vision, understanding, clarity and agility.^10,11^

## Curiosity – Is It the Driver to Seeing Things Differently?

Surely it is. Curiosity entails the quality of inquisitive thinking. Inquisitive thinking inevitably leads to broadening the outlook and to expanding the horizon, seeing other possibilities and linkages. Curiosity drives the desire to make sense of the unknown, overcome uncertainties and achieve coherence, all of which ultimately results in seeing the bigger picture and thus avoiding the trap of reaching “well-known solution that are neat, plausible, and wrong” (paraphrasing Mencken^12^).

True curiosity is a matter of mindset and worldview, it entails a willingness to deeply engage and an ability to painstakingly work through the seemingly contradictory and chaotic pathways inherent in a puzzling *wicked* problem.^9,13^ Curiosity is primarily about one’s personal quest to seek meaning and understanding rather than the ­*a-priori* pursuit of Menckenian false certainty and truth. Failure to challenge one’s mental models or mindset has the inherent risk of perpetuating self-confirming inferences that allow harmful beliefs and behaviours to undermine the emergence of novel ideas and solutions to otherwise intractable – VUCA world – problems.^13^

## Leadership – Showing the Way to Find out Together

Leaders clearly need to change the way they think, as it indeed transforms one’s mind models for doing one’s work. This is particularly important for people with leadership responsibilities. Their challenge is to constantly remain cognisant about “[facilitating]* the necessary adaptive work that needs to be done by the people connected to the problem*.”^14^ Heifetz’s perception of leadership focuses on engagement and an acknowledgement that engaging people in the process of problem-solving requires the permission to try out different solutions. Some will fail – failure is part of problem-solving and must be anticipated as an inevitable part of the process to success. True leaders regard failures not as *having failed* but rather as opportunities to *facilitate collective learning*.

While it may be true that most “*people make sense of the world given what they know*” and that “*without a compelling rationale,* [they] *tend to hold on to established mental models* [to] *avoid the disruption of seeing the world in radical new ways*,”^2,15^ these are clearly not the attributes we look for in leaders that have to deal with *wicked*problems. Policy problems are indeed “*entangled in complex social, economic, political and institutional contexts*” that can only be fully appreciated and managed applying a *systems and complexity thinking* framework. It is the *systems and complexity thinking* framework that allows us to work towards a (as far as possible) comprehensive understandings of the issues in terms of identifying the full range of feedback and the consequences of its associated embedded time-delays.^13^ It would indeed be an oxymoron to attribute any particular approach to solving systemic problems a *panacea*, nevertheless, for complex problems *systems and complexity thinking* is as close to one as one can get.

Leaders require a deep – indeed “*academic*” – understandings of the nature of the problems and the veracity of various problem-solving approaches. Intellectual disengagement and/or neglect cannot be justified – here in the context of bureaucrats and policy-makers – on the ground that “*There is more work to*[be done] i*n articulating system thinking and in demonstrating its policy utility, including developing practical tools and real-world case studies that show how systems approaches can impact outcomes*.”^2^ A curious person would – even with only a cursory look at the literature – identify plenty of examples of successful translation of *systems and complexity thinking* across a wide range of health and health policy problems.^6,16-20^

Indeed, leaders are expected to not only communicate the complex nature of problems^21^ but also to be active participants in the problem-solving process – they must see themselves as also being “*connected to the problem*” and join in in doing “*the necessary adaptive work*.”^14^

## Curiosity Ought to Be a Prerequisite for Becoming a Bureaucrat and/or Policy-Maker

As Sterman^13^ has emphasised effective change in complex system requires creativity, and curiosity is a key driver of creativity. As Haynes et al^2^ observed there is some hope that – given more time and even more patience – some receptive people within the bureaucracy might succeed in promoting *systems and complexity thinking* change – and thereby lead transformational change within the bureaucracy (Figure) – despite its challenges:

**Figure:**
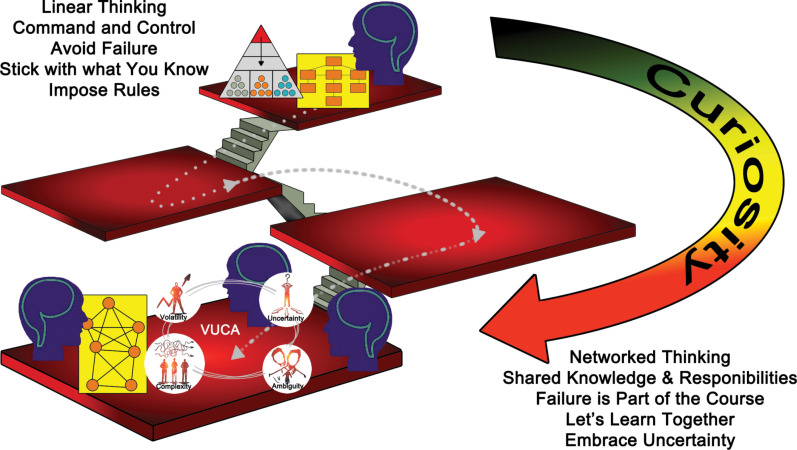
Curiosity – the PANACEA of Transformational Leadership Change in the Bureaucracy.

*Perhaps most importantly, despite the cogitative challenges mentioned above, systems thinking seemed to be helping policy partners to reconceptualise health problems and contexts, goals, potential policy solutions, and approaches to developing those solutions, including prevention risk factors, outcomes and indicators, measures and roles*.

However, it is worrying that policy-makers “*express* [little] *excitement about abstract theories or principles, but* [rather] *about applying systems thinking to specific concerns in their local contexts.”* It is questionable if one truly can apply *systems and complexity thinking* meaningfully without a deep understanding of its foundations.

*Systems and complexity thinking* is *not just another tool* to be pulled out of the box to throw at a problem. *Systems and complexity thinking* is – in a broader sense – a way of being, namely deeply interested in understanding the highly interconnected and interdependent nature of an issue affecting our life and work. This stands in stark contrast to the authoritarian expert mode “*that dulls creativity and stunts the development of the skills needed to catalyze effective change in complex systems.*”^13^ Systemic change is transformative, let’s hope it will become normalised^22^ and common practice (Figure).

Hence, *system and complexity thinking* is, contrary to what Haynes et al^2^ state in their “summation for the public reader,” neither “*overwhelming and hard* [nor difficult] *to use practically*.” Such a view is as much misleading as self-defeating.

## Ethical issues

Not applicable.

## Competing interests

Author declares that he has no competing interests.

## Author’s contribution

JPS is the single author of the paper.
